# Safety and Acceptability of the PrePex Device When Used in Routine Male Circumcision Service Delivery During Active Surveillance in Zimbabwe

**DOI:** 10.1097/QAI.0000000000000721

**Published:** 2016-05-24

**Authors:** Webster Mavhu, Karin Hatzold, Getrude Ncube, Sinokuthemba Xaba, Ngonidzashe Madidi, Jo Keatinge, Efison Dhodho, Christopher A. Samkange, Mufuta Tshimanga, Tonderayi Mangwiro, Owen Mugurungi, Emmanuel Njeuhmeli, Frances M. Cowan

**Affiliations:** * Centre for Sexual Health and HIV/AIDS Research (CeSHHAR), Harare, Zimbabwe;; † Population Services International, Harare, Zimbabwe;; ‡ Ministry of Health and Child Care, Harare, Zimbabwe;; § United States Agency for International Development, Harare, Zimbabwe;; ‖ University of Zimbabwe College of Health Sciences, Harare, Zimbabwe;; ¶ United States Agency for International Development, Washington, DC; and; # University College London, London, United Kingdom.

**Keywords:** PrePex, male circumcision, safety, acceptability, Zimbabwe

## Abstract

**Background:**

Male circumcision devices have the potential to accelerate voluntary medical male circumcision roll-out, with PrePex being one promising device. Here, we present findings on safety and acceptability from active surveillance of the implementation of PrePex among 1000 males circumcised in Zimbabwe.

**Methods:**

The first 1000 men consecutively circumcised using PrePex during routine service delivery were actively followed up. Outcome measures included PrePex uptake, attendance for postcircumcision visits, and adverse events (AEs). A survey was conducted among 500 consecutive active surveillance clients to assess acceptability and satisfaction with PrePex.

**Results:**

A total of 2156 men aged 18 years or older were circumcised across the 6 PrePex active surveillance sites. Of these, 1000 (46.4%) were circumcised using PrePex. Among them, 4 (0.4%) self-removals that required surgery (severe AEs) were observed. Six (0.6%) removals by providers (moderate AEs) did not require surgery. A further 280 (28%) AEs were mild or moderate pain during device removal. There were also 12 (1.2%) moderate AEs unrelated to pain. All AEs resolved without sequelae. There was high adherence to follow-up appointments, with 97.7% of clients attending the scheduled day 7 visit. Acceptability of PrePex was high among survey participants, 93% indicated willingness to recommend the device to peers. Of note, 95.8% of respondents reported experiencing pain when the device was being removed. Additionally, 85.2% reported experiencing odor while wearing the device or during removal.

**Conclusions:**

Active surveillance of the first 1000 men circumcised using PrePex suggests that the device is both safe and acceptable when used in routine service delivery.

## INTRODUCTION

Fourteen African countries are currently accelerating voluntary medical male circumcision (VMMC) roll-out.^1–5^ Modeling studies conducted between 2009 and 2011 suggested that circumcising males aged 15–49 years to reach 80% coverage within 5 years in these countries, and maintaining this coverage thereafter, could avert 3.4 million new HIV infections within 15 years and yield treatment and care savings of US$16.5 billion.^2,3^ The modeling also suggested that the faster coverage of VMMC can be achieved, the greater the number of infections averted.^3^ Male circumcision devices have the potential to accelerate VMMC roll-out by making the procedure easier, quicker, and more widely accessible.^4,6^ One promising device for VMMC is PrePex, which works by compressing the foreskin between a ring and an elastic band, leading to distal tissue necrosis.^7^

Following results from PrePex device studies in Rwanda,^8,9^ additional research with the device was conducted in Zimbabwe to establish its safety, efficacy, and acceptability among providers and clients.^10–12^ Data from these studies contributed to the prequalification of PrePex by the World Health Organization (WHO) for use in adults aged 18 years or older.^13^

In addition to the criteria defined in the *Framework for Clinical Evaluation of Devices for Male Circumcision*,^14^ WHO also outlined an evaluation series that each country considering introduction of male circumcision devices should complete. The WHO Technical Advisory Group on Innovations in Male Circumcision further recommended active follow-up of the first 1000 clients circumcised using a new device.^15^ This active follow-up should take place within the context of routine service delivery.^15^

Zimbabwe is among the first countries to actively follow-up clients circumcised using PrePex during routine service delivery (ie, under programmatic rather than research conditions). Here, we present findings on safety and acceptability of the device among the 1000 males circumcised using PrePex during active surveillance.

## METHODS

Data presented here are from (1) active surveillance of the first 1000 men consecutively circumcised using PrePex during routine service delivery and (2) a survey conducted among 500 consecutive active surveillance clients to assess acceptability and satisfaction with PrePex.

### Active Surveillance

Between March 31, 2014, and June 2, 2014, PrePex circumcisions were conducted at 6 VMMC clinics in 4 of Zimbabwe's 10 provinces (Harare n = 2 clinics, Bulawayo n = 2 clinics, Manicaland n = 1 clinic, and Mashonaland West n = 1 clinic). Three of the 6 VMMC clinics had previously been sites for PrePex device studies.^10–12^ VMMC staff at the 6 sites were trained in active surveillance standard operating procedures and data collection tools. In addition, male researchers were deployed at 4 of the 6 sites specifically for data collection (n = 2 sites in Harare and n = 2 sites in Bulawayo). At remaining sites, data were collected by trained VMMC staff.

### Outcome Measures

Outcome measures for the active surveillance included (1) percentage of men seeking VMMC who chose PrePex over the surgical procedure, (2) percentage of PrePex clients failing to return to the clinic for scheduled visits on days 7, 14, and 49, (3) percentage of PrePex clients returning to the clinic for each scheduled visit after receiving reminders, and (4) percentage of adverse events (AEs). AEs were classified according to the surveillance standard operating procedures and PrePex AE guidelines. Early removals requiring surgery were classified as severe AEs, the rest were recorded as moderate AEs. Pain was assessed using a visual analog scale ranging from 0 (no pain) to 10 (highest pain) and measured at different time points—at device application, while wearing PrePex, and at removal.

### Client Active Follow-up

During active surveillance, the first 1000 men aged 18 years or older seeking VMMC at the 6 clinics who opted and were eligible for circumcision using PrePex were actively followed up for their PrePex circumcision and postoperative wound care. Clients provided their mobile phone numbers and home addresses so they could be tracked in the event that they missed a scheduled postcircumcision visit. In addition, clients were instructed to return to the clinic outside scheduled visits if they experienced any AEs or complications with the device.

For active surveillance, if a client failed to attend day 7 scheduled visit, clinic staff made at least 3 attempts to contact him by phone and made at least 2 home visits, if necessary. If a client failed to attend a scheduled visit after removal of the device (days 14 or 49), clinic staff made at least 3 attempts to contact him by phone but no home visits were conducted. All missed appointments were rescheduled to a time that was convenient to the client and consistent with clinic hours. If the client was unable to attend, he was assessed over the phone using a standard set of questions to determine the presence of AEs, the extent of wound healing, and any wound care practices. AEs were documented at each visit. All attempts to contact the client were recorded on a contact log as were reasons reported for missing the scheduled visit. Routine VMMC monitoring data as per national guidelines were also collected.

### Acceptability and Satisfaction Survey

Five hundred consecutive men who had undergone PrePex male circumcision, attending for the second scheduled visit at day 14, were asked to take a short interviewer-administered structured questionnaire. Questionnaire items explored satisfaction with the procedure and perceptions of pain and odor. Survey respondents were asked to indicate pain severity on a numerical scale ranging from 0 (no pain) to 100 (highest pain). They were also asked to indicate discomfort with odor on a numerical scale ranging from 0 (no odor) to 100 (highest odor). Additionally, they were asked to indicate satisfaction with PrePex circumcision outcome on a numerical scale ranging from 0 (no satisfaction) to 100 (highest satisfaction). The questionnaire was programmed using Entryware software, and tablets were used for data collection. Skip instructions and mandatory data fields were used to ensure data validity, consistency, and completeness. The questionnaire was administered by the male researchers in either Shona or Ndebele, Zimbabwe's dominant indigenous languages, also spoken and understood by smaller ethnic groups.

### Data Processing and Analysis

Active surveillance data from the 6 sites were entered into a database and analyzed to ascertain the percentage of men seeking VMMC who chose PrePex over the surgical procedure, the percentage of PrePex clients failing to return to clinic (days 7, 14, and 49), the percentage of PrePex clients returning to the clinic for each scheduled visit after receiving reminders, and percentage of AEs.

Questionnaire data were downloaded into an Access database. Completeness and consistency checks were performed. Any anomalies in the data were verified and corrected. Descriptive analyses of key variables were performed. Data were analyzed using Stata 12 (Stata Corp, College Station, TX).

### Ethical Considerations

This acceptability and satisfaction study was approved by the Medical Research Council of Zimbabwe, the Research Council of Zimbabwe, the Ethics Board of Population Services International, and the University College London Ethics Committee. All participants provided written informed consent.

## RESULTS

### Active Surveillance: PrePex Eligibility and Uptake

Between March 31, 2014, and June 2, 2014, a total of 2156 men aged 18 years or older were circumcised across the 6 PrePex active surveillance sites. Of these, 1000 (46.4%) were circumcised using PrePex and the remainder (53.6%) using conventional surgery. Here, we present only results of men circumcised using PrePex.

Uptake of VMMC using PrePex was relatively higher at the 2 Bulawayo sites as compared with the 2 Harare clinics (66.7% and 23.9%, respectively, *P* < 0.01) (Table 1). Across the 6 sites, 72 of 1072 (6.7%) men who indicated willingness to be circumcised using PrePex were found to have contraindications for this particular method. These included phimosis, tight foreskin, paraphimosis, hypospadias (n = 57, 79.2%), balanitis, sexually transmitted infections (n = 11, 15.3%), hemophilia, and uncontrolled hypertension (n = 2, 2.8%). In addition, in 2 cases (2.8%), the available PrePex device sizes were too small to fit the client's anatomy.

**TABLE 1.**
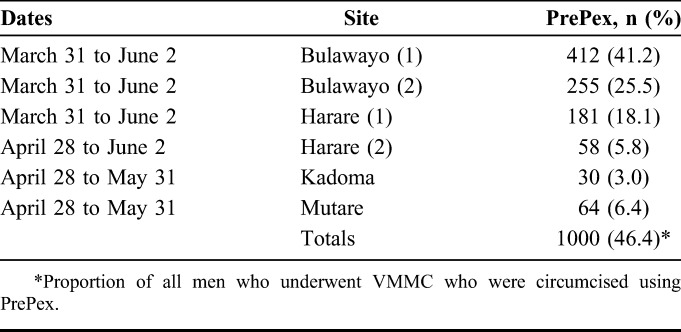
Uptake of PrePex VMMC Across the 6 Sites

### Active Surveillance: Frequency and Outcomes of Follow-up

There was good adherence to follow-up appointments with 977 (97.7%) clients returning to the VMMC site for the scheduled day 7 visit without any reminders being sent. All 23 (2.3%) men who did not return to the VMMC site for the visit were successfully tracked. Of these, 17 (73.9%) returned to the VMMC clinic on day 8 after at least 2 text message reminders and 2 call attempts. All cited work commitment as their reason for missing the scheduled day 7 visit. Three (13%) returned on day 9, of whom one was followed at home. One client (4.3%) returned to the VMMC site on day 10 and 2 clients (8.7%) on day 14 after at least 2 text message reminders and 2 call attempts. The 3 clients who presented on days 10 and 14 still had the device in place and the foreskin attached.

Clients who reported to the clinic on days 9–14 were either long distance truck drivers or individuals who were working in remote parts of the country. A total of 198 (19.8%) clients circumcised using PrePex failed to attend their scheduled visit on day 14, and about half of (495, 49.5%) failed to attend for review on day 49, despite 3 text message reminders/call attempts.

### Safety: AEs

Table 2 summarizes AEs that occurred during the active surveillance. A total of 328 of the 1000 (32.8%) AEs were observed. Five (0.5%) were self-removals, 4 of which required surgery (severe AEs). Six (0.6%) early removals by providers (moderate AEs) did not require surgery. A further 183 (18.3%) and 97 (9.7%) AEs were mild and moderate pain during device removal, respectively. There were also 12 (1.2%) moderate AEs unrelated to pain—swelling (n = 6), bleeding (n = 3), and infection (n = 3). All AEs resolved without sequelae. The AEs are described in detail below.

**TABLE 2.**
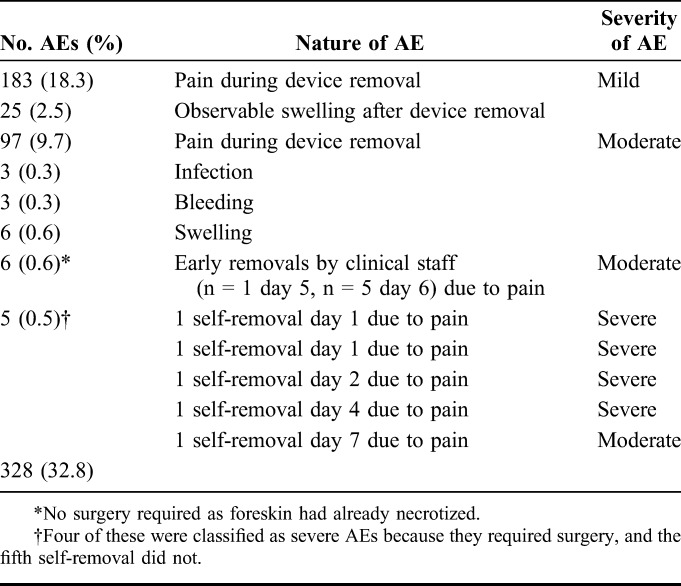
Number, Nature, and Severity of PrePex VMMC AEs

Five (0.5%) men removed the device themselves due to pain. Four of the 5 self-removals occurred on days 1, 2, and 4. In all 4 cases, dorsal slit circumcision was performed 24–48 hours after self-removal. The fifth self-removal, classified as moderate, occurred on day 7. The client used a kitchen knife to remove the device plus the dead foreskin, and no further intervention was required.

Six (0.6%) early removals by providers occurred on days 5 and 6. In all cases, the foreskin had already necrotized and was easy to remove. One of these early removals was due to pain as a result of the device being kicked (day 6) during a fight. The device displaced and was removed without further complications.

### Survey: Acceptability of PrePex

**FIGURE 1.**
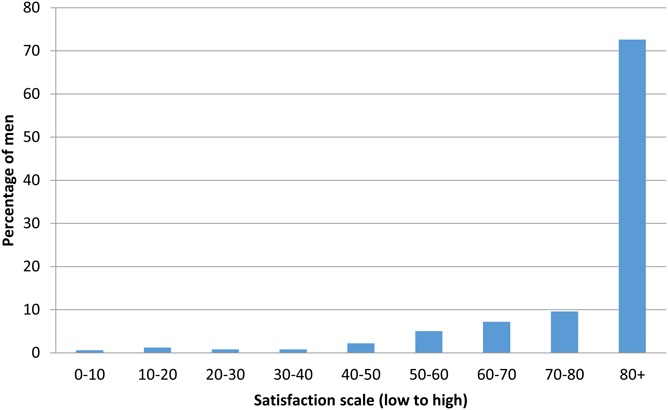
Satisfaction with PrePex device outcome (n = 465).

Between April and June 2014, 500 participants who had undergone PrePex male circumcision completed an interviewer-administered questionnaire when they attended day 14 review visit. No one declined to take part in the study. Acceptability of the device was high with 465 (93%) men, indicating that they would recommend circumcision using PrePex to their peers. Of these, 418 (89.9%) ranked their satisfaction with PrePex outcome to be at least 60% on the numerical scale (Fig. 1).

### Survey: Perceptions of Pain

**FIGURE 2.**
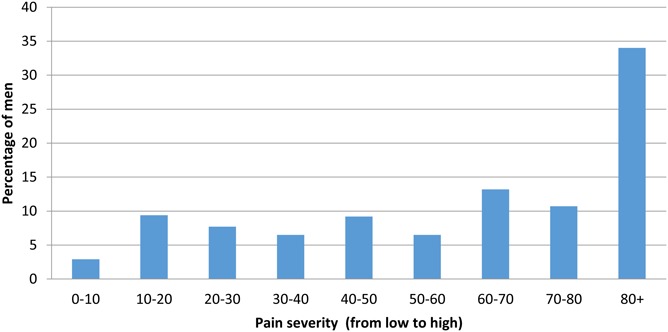
Distribution of pain severity when device was removed (n = 479).

A total of 464 (92.8%) respondents reported experiencing pain while wearing the device (days 2–4). There were no differences by study site (data not shown). Furthermore, 479 (95.8%) respondents reported experiencing pain when the device was being removed, with 274 (57.2%) estimating their pain severity to be 60%–100% (Fig. 2). Of these 479 respondents, 111 (23.2%) stated that they would have opted for surgical circumcision instead of PrePex if they had known the extent of pain. Forty-five respondents (9.4%) stated that they would have decided not to be circumcised at all. Among 34 respondents who indicated that they would not recommend PrePex to their peers, 30 (88.2%) ranked their pain during device removal to be at least 60% on the numerical scale. Among the 465 respondents who indicated that they would recommend PrePex to their peers, 264 (56.8%) (*P* ≤ 0.01) ranked their pain similarly at 60% or above.

### Survey: Perceptions of Odor

**FIGURE 3.**
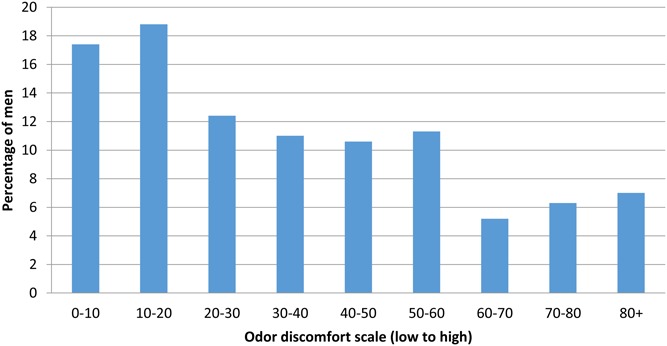
Extent to which made the respondent uncomfortable (n = 426).

A total of 426 (85.2%) respondents reported experiencing odor while wearing the device (days 3–6) or during removal and that the odor made them feel uncomfortable (Fig. 3). Of these, 41 (9.6%) indicated that they would have chosen surgical circumcision over PrePex if they had known about the odor. Six (1.4%) stated that they would have decided not to be circumcised at all. Among 34 respondents who indicated they would not recommend PrePex to their peers, 16 (47.1%) ranked their discomfort with odor to be at least 60% on the numerical scale. Among the 465 respondents who would recommend PrePex to their peers, 135 (29%) (*P* = 0.04) ranked their discomfort with odor similarly at 60% or above.

## DISCUSSION

We report active surveillance of the first 1000 males circumcised using the PrePex device during routine service delivery in Zimbabwe. Four (0.4%) self-removals that required surgery (severe AEs) were observed. Six (0.6%) removals by providers (moderate AEs) did not require surgery. Separating out moderate AEs related to pain, 12 of the 1000 (1.2%) moderate AEs were observed. This low rate of severe or moderate AEs is comparable with that observed in PrePex device research studies.^8,9,16–19^ Active surveillance data suggest that PrePex can be safely used with adult males in routine VMMC program roll-out. The device's safety, efficacy, and acceptability within routine service delivery will be more accurately quantified in a larger (n = 9000) follow-up passive surveillance that is being conducted in the same study population.

There were 108 (10.8%) pain-related moderate or severe AEs. The experience of pain was also reported in the survey where 464 (92.8%) respondents reported experiencing pain while wearing the device and 479 (95.8%) when the device was being removed. Surprisingly, despite reporting experiencing at least 60% pain severity during device removal, 56.8% would still recommend PrePex to their peers. This suggests that the pain experienced during device removal is short-lived and is probably within clients' expectations.^8,9,16–21^ Nonetheless, the fact that 45 of the 479 respondents (9.4%) who experienced pain during device removal felt that they should not have been circumcised at all warrants consideration. Additionally, and as found in other studies,^16–18,20^ survey participants reported experiencing odor while wearing the device and at removal, and this odor resulted in discomfort.

Concerning these 2 issues, providers need to help clients manage both pain and odor throughout the procedure, particularly as a few men reported that the pain and odor were intolerable to the extent that they would not recommend circumcision using PrePex to peers. Study findings resulted in a change to the analgesic protocol for PrePex VMMC clients while wearing the device (from paracetamol to ibuprofen) and at device removal (application of local anesthetic cream before device removal). Furthermore, the program intensified counseling messages and strengthened the information and education materials to minimize the risk of self-removal by the clients. Materials also addressed the issue of pain and odor plus how to minimize these while wearing the device.

Uptake of PrePex during the active surveillance (46.4%) was lower than that of conventional surgery (53.6%). Our findings are in agreement with previous studies that have shown that not every client will opt to be circumcised using PrePex.^17,22^ It should be noted that no specific campaigns or interpersonal communication sessions had been developed specifically for PrePex and most men presented at the VMMC clinics not knowing about the new procedure. It is assumed that wider knowledge about the device and extending the eligibility criteria to include adolescents aged 13–17 years will likely increase PrePex uptake. Nonetheless, there is need to run specific demand creation campaigns to increase both knowledge and uptake of circumcision using PrePex.^4^

Although uptake of PrePex was lower than that of conventional surgery, satisfaction with the device was high among clients who did opt for it, with 93% of survey respondents indicating that they would recommend the device to their peers, and 89.9% ranking their satisfaction with the outcome to be at least 60%. Other PrePex studies and those evaluating the ShangRing also found high levels of satisfaction with these devices.^9,18,20,23,24^ To maintain these high levels of satisfaction with circumcision in general and device-led circumcision in particular, programs will need to be carefully supervised and monitored to ensure (1) a good cosmetic result and (2) that AEs, including experiencing pain, are prevented.^25^

The high adherence to the scheduled day 7 appointment recorded in the active surveillance (98%) is encouraging and is similar to that observed in other PrePex device studies.^8,18^ However, a decline in the follow-up rate was observed on days 14 (80%) and 49 (50%), despite text message reminders and call attempts. A study conducted in Kenya, South Africa, Tanzania, and Zimbabwe among surgical VMMC clients also found that follow-up decreases with each scheduled postcircumcision visit.^26^ Given the suboptimal adherence to day 49 follow-up appointment and subsequent assurances by clients reached by phone that complete healing had been achieved, it may be necessary to review the postcircumcision follow-up protocol. Of note, 2 clients who reported to the VMMC site later than the scheduled day 7 visit indicated that they were in remote parts of the country and local health facilities were unable to remove the device. Further decentralization of VMMC services should ensure that lower health facilities are capacitated to assist VMMC clients who present with challenges associated with both device- and surgical-related issues.

### Limitations

A potential limitation of the findings presented here is that we do not have information to explain differences in PrePex uptake between the Bulawayo and Harare sites. Also, only the men who returned for day 14 visit were interviewed, and therefore, they may not be representative of the entire population that was circumcised using PrePex. In addition, we did not survey those men who were circumcised conventionally. A follow-up qualitative study is exploring in-depth several issues among men circumcised using PrePex and conventionally.

## CONCLUSIONS

We successfully followed up the first 1000 men circumcised using PrePex during routine service delivery and surveyed 500 of them to determine device safety as well as acceptability and satisfaction. We found that PrePex is both safe and acceptable when used in routine service delivery. The device therefore has the potential to facilitate widespread scale-up of safe VMMC in sub-Saharan Africa. However, there is need to increase awareness about the device and to train healthcare providers in remote sites to handle follow-up of men after circumcision.
